# First Report of Root-knot Nematode, *Meloidogyne Graminicola* on *Brassica Juncea* in China

**DOI:** 10.2478/jofnem-2022-0044

**Published:** 2022-10-19

**Authors:** X. H. Lu, G. S. Solangi, J. L. Huang, L. P. Qin, Z. M. Liu

**Affiliations:** 1 Guangxi Key Laboratory of Biology for Crop Diseases and Insect Pests, Key Laboratory of Green Prevention and Control on Fruits and Vegetables in South China Ministry of Agriculture and Rural Affairs, Institute of Plant Protection, Guangxi Academy of Agricultural Sciences, Nanning 530007, Guangxi China; 2 Department of Entomology, Shaheed Zulfiqar Ali Bhutto Agricultural College, Dokri 77060, A constitute College of Sindh Agriculture University, Tandojam, Pakistan

**Keywords:** *COII*, *Meloidogyne graminicola*, mustard, pathogenicity, host-parasitic relationship, rDNA

## Abstract

In southern China, the staple food rice (*Oryza sativa*) field is commonly rotated with brown mustard *Brassica juncea*. Root-knot nematodes (RKNs) are a major threat to rice production. From 2019 to 2021, *B. juncea* in 56 fields from 26 counties in Guangxi Province were observed with symptoms of leaf yellowing, stunting, and several hook-shaped galls on the roots. Females and egg masses of *Meloidogyne* sp. were found within the galls. The females, males, and second-stage juveniles (J2s) were collected, and identified with morphological and molecular characteristics and female perineal patterns. *Brassica juncea* was transplanted in pots and a pathogenicity test was conducted to confirm the species as *Meloidogyne graminicola*. In China, this is the first record of a natural infection of mustard with *M*. *graminicola*, and this finding has great importance for Chinese mustard production, since this nematode may damage mustard plants and become an additional problem for this crop.

Nowadays Brassicaceae vegetables are highly diversified in Mediterranean Europe, Asia, and North America (Al-Shehbaz, 2011). Mustard (*Brassica juncea*) is an important crop with a long history of cultivation in China (Chen *et al*., 2013), where it is used as both an oilseed and a vegetable crop. In China, the area under cultivation of mustard is 7.03 million ha (Jannat *et al*., 2022), and production for the year 2021 was 14.70 million metric tons (WAP, 2022). However, mustard has become an important vegetable crop in China from the point of view of export utility, and Guangxi is an important mustard production province.

China is a major importer and exporter in the world rice market, and it is expected that the country will increase its rice production in the near future. Rice is one of the prominent cereal crops in China, and about 65% of Chinese people rely on rice. Nearly 95% of the rice grown in China is produced under the traditional puddled transplanted conditions that are typically found in China and characterized by prolonged periods of flooding during cultivation (Nie and Peng, 2017). One of the major economic constraints for rice production is the infection caused by rice-parasitic nematodes.

Root-knot nematode is a pest of international importance to commercial rice cultivation around the world and it poses one of the great concerns about yield loss due to nematode infestation in rice and wheat crops under the rice–wheat cropping system. The large host range of *Meloidogyne graminicola* and its ability to survive for long periods in environments with low oxygen render its control very difficult. For these reasons, adequate phytosanitary measures are important.

Rice RKN *M. graminicola* (Nematoda: Meloidogynidae) was first isolated from the roots of barnyard grass *Echinochloa colonum* L. in Baton Rouge, LA, reported by Golden and Birchfield (1965). *M. graminicola* is one of the most prevalent RKNs and is a major threat to staple food rice (*Oryza sativa*) production (Mantelin *et al*., 2017).

In China, *M*. *graminicola* was first discovered on Welsh onion (*Allium fistulosum*) in Hainan Province (Zhao *et al*., 2001). The nematode was then discovered on rice in China in Fujian, Hunan, Henan, Hubei, Zhejiang, Jiangxi, and Sichuan Provinces (Song *et al*., 2017; Xie *et al*., 2019). The incidence of the disease is severe in Hunan. Disease incidence exceeded 85% in infected paddy fields (Song *et al*., 2017). In southern China, the rice field is commonly rotated with brown mustard (*B. juncea*), also called Indian mustard or Chinese mustard.

In July 2016, *M. graminicola* was detected for the first time in the EPPO region in several rice fields in Northern Italy, and accordingly the EPPO Secretariat decided to add this nematode to the EPPO Alert List. In 2017, it was also found in Lombardia. *M. graminicola* infests many plant species belonging to different families (mainly Poaceae but also Asteraceae, Cucurbitaceae, Fabaceae, and Solanaceae) that include cultivated plants of economic importance to the EPPO region (EPPO, 2016).

A survey of the plant-parasitic nematode infestation of rice fields was conducted during 2018 to 2019. The rice plants with leaf chlorosis were found in 206 fields in 67 counties in Guangxi, China around 30 d after transplanting (Luo *et al*., 2020) but mustard was not recorded as a host plant. From 2019 to 2021, *B. juncea* in 56 fields from 26 counties in Guangxi Province were observed with symptoms of leaf yellowing, stunting, and several hook-shaped galls on the roots. Females and egg masses of *Meloidogyne* sp. were found within the galls. This *Meloidogyne* sp. was maintained in a greenhouse on mustard plants. Morphological, morphometrical, and molecular characterization of this isolate was made from the mustard culture.

The females, males, and second-stage juveniles (J2s) were collected, and identified with morphological and genetic characteristics (Xie, 2005). The perineal patterns of females (Fig. 1) were prepared following Hunt and Handoo (2009) and observed under a light microscope as ovoid shapes. Striae were smooth and fine, and broken by short and irregular striae in the dorsal part of the pattern, while phasmids were small but distinct and closely spaced. Measurements of females (*n =* 10) resulted in the following observations: stylet length, 12.04 ± 0.34 (10.62–14.11) mm; dorsal gland orifice (DGO), 4.24 ± 0.08 (3.91–4.73) mm; vulva slit length, 23.39 ± 0.90 (19.18–27.23) mm; and interphasmidial distance, 15.15 ± 1.23 (9.82–19.53) mm. Measurements of males (*n* = 10) resulted in the following observations: body length, 1,288.20 ± 185.47 (1,020.34–1,601.25) mm; body width, 40.04 ± 2.19 (36.49–42.01) mm; stylet length, 16.36 ± 0.66 (15.21–17.42) mm; DGO, 3.85 ± 0.30 (3.54–4.28) mm; and spicule length, 30.53 ± 1.08 (28.69–32.10) mm. Measurements of J2s (*n* = 20) resulted in the following observations: body length, 452.65 ± 17.77 (413.81–481.53) μmm; body width, 15.86 ± 1.02 (13.92–17.41) mm; a, 28.66 ± 2.19 (24.60–32.52); c, 6.08 ± 0.34 (5.72–6.69); stylet length, 12.54 ± 0.70 (11.23–13.84) mm; DGO, 3.59 ± 0.62 (2.10–4.68) mm; anterior end to excretory pore, 76.65 ± 2.54 (71.75–79.60) mm; tail length, 74.58 ± 3.24 (69.82–78.93) mm; and hyaline tail length, 21.27 ± 2.29 (18.20–25.52) mm. The morphology and morphometrics of this species confirmed its identity as *M. graminicola*, which has been described previously (Golden and Birchfield, 1965; Zhao *et al*., 2001).

**Figure 1:**
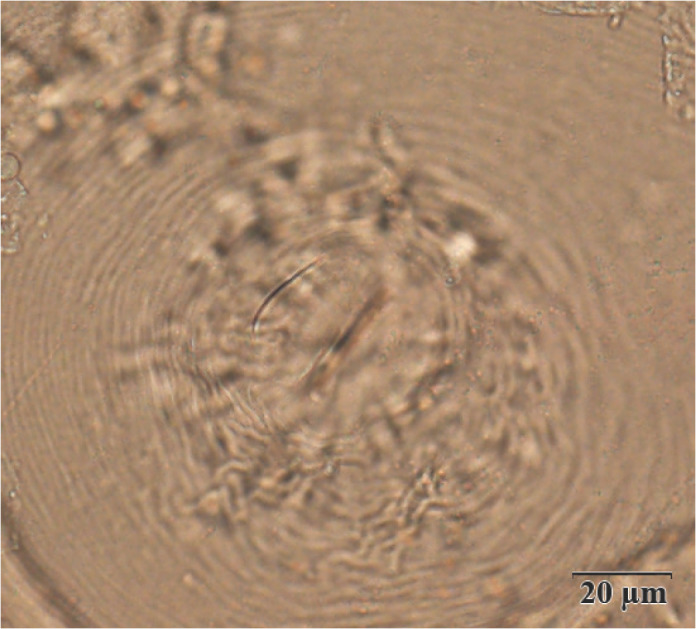
Perineal patterns of *Meloidogyne graminicola*.

In addition, sequence analyses were performed to confirm species identification. DNA was individually extracted from four J2s using the method of Qiu *et al*. (2016). For the amplification of the D2– D3 region of the 28S rRNA, the forward D2A (5¢-ACAAGTACCGTGAGGGAAAGTTG-3¢) and the reverse D3B (5¢-TCGGAAGGAACCAGCTACTA-3¢) primers were used (De Ley *et al*., 1999). The ITS1-5.8S-ITS2 rDNA region was amplified with V5367 (5¢-TTGATTACGTCCCTGCCCTTT-3¢) and 26S (5¢-TTTCACTCGCCGTTACTAAGG-3¢) (Vrain *et al*., 1992). The region of the mitochondrial genome between the cytochrome oxidase subunit II (*COXII*) and 16S rRNA mitochondrial DNA (mtDNA) gene was amplified using primers C2F3 (5¢-GGTCAATGTTCAGAAATTTGTGG-3¢) (Powers and Harris, 1993) and MRH106 (5¢-AATTTCTAAAGACTTTTCTTAGT-3¢) (Stanton *et al*., 1997).

The length of the D2/D3 region of *M. graminicola* was 772 bp (GenBank Accession Nos. MN648521 and MN648522), which had 99.48% to 100% similarity with those from Fujian, China (MT159670) and USA (JN157844). The ITS1-5.8S-ITS2 rDNA region yielded a PCR fragment of 617 bp (MN636702 and MN636711), which had 99.67% to 99.17% similarity with those from Taiwan, China (KJ572383), and Vietnam (MG773553). However, *COXII* 665 bp (ON840000) had 100% similarity with those from China (KM111533) and India (OK245416).

*Brassica juncea* var. *foliosa* Bailey (sprout mustard), Guitian cultivar seed was obtained from Vegetable Research Institute, Guangxi Academy of Agricultural Sciences, Nanning, Guangxi, China. The seeds were sown in trays filled with commercial substrate, which was obtained Changchun Saishi Agricultural Development Company, Ltd., to conduct a pathogenicity test. Twelve 3-wk-old seedlings were transplanted into 12 pots (one per pot). The pot size was 22 cm wide × 15 cm high, and it was filled with sterilized soil (2 kg) and placed in the glasshouse at 25 ± 2°C with 65 ± 5% relative humidity. Nine plants were inoculated with 500 J2s per pot hatched from the egg masses of the original population of *M. graminicola*, and the other three non-inoculated plants served as a control. After 9 weeks, hook-shaped galls were observed on the roots (Fig. 2), with J2, and eggs were found within the galls. Morphological characteristics of the nematodes isolated from inoculated roots were identical to those in the field samples. Reproductive factor (nematode final population density/initial population density) was 6.52. No galls were observed in the control plants. These results confirmed the nematode’s pathogenicity on *B. juncea*. In China, this is the first record of a natural infection of *B. juncea* var. *foliosa* Bailey (sprout mustard), Guitian cultivar with *M. graminicola*, and this finding has great importance for Chinese mustard production, since this nematode may damage mustard plants and become an additional problem for this crop.

**Figure 2:**
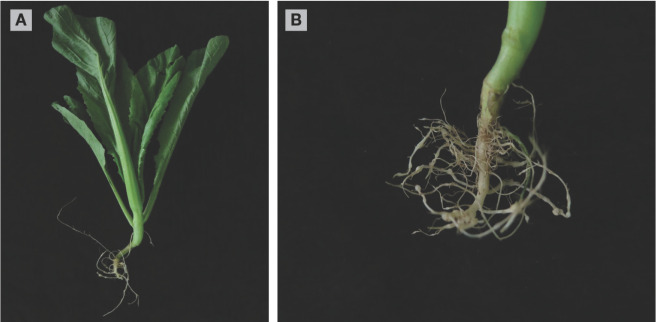
Roots of *Brassica juncea* showing galls **(A)** 42 d and **(B)** 63 d post inoculation induced by *Meloidogyne graminicola*.

## Acknowledgments

This research was funded by the National Natural Science Foundation of China (31860492), Natural Science Foundation of Guangxi (2020GXNSFAA297076), and Guangxi Academy of Agricultural Sciences Fund, China (2021YT062, 2021JM14, 2021ZX24).
